# L-serine combined with carboxymethyl chitosan guides amorphous calcium phosphate to remineralize enamel

**DOI:** 10.1007/s10856-023-06745-z

**Published:** 2023-09-02

**Authors:** Yinghui Wang, Shuting Zhang, Peiwen Liu, Fan Li, Xu Chen, Haorong Wang, Zhangyi Li, Xi Zhang, Xiangyu Zhang, Xu Zhang

**Affiliations:** 1 School and Hospital of Stomatology, Tianjin Medical University, Tianjin, 300070 China; 2 Department of stomatology, Economic and Technological Development Zone, No.7 people’s hospital of Zhengzhou, No. 17, Jingnan 5th Road, Zhengzhou City, Henan Province 450003 China; 3 Department of Stomatology, the Fifth Central Hospital of Tianjin, No. 41, Zhejiang Road, Tanggu, Binhai New District, Tianjin, 300450 China; 4 Institute of Stomatology, Tianjin Medical University, Tianjin, 300070 China

## Abstract

The aim of this study is to investigate a robust and stable calcium-phosphorus system to remineralize human early enamel caries lesions with nanocomplexes of carboxymethyl chitosan/L-serine/amorphous calcium phosphate (CMC-Ser-ACP) to develop an effective method for mimicking the amelogenin (AMEL) mineralization pattern through ACP assembly. A CMC-Ser-ACP nanocomplex solution was first synthesized by a chemical precipitation method, and then 1% sodium hypochlorite (NaClO) was added to induce ACP phase formation. The morphologies of the nanocomplexes were characterized by transmission electron microscopy (TEM), and zeta potential analysis and Fourier transform infrared spectroscopy (FTIR) were performed to detect surface charge and functional group changes. The subtle changes of the demineralized enamel models induced by the remineralization effect were observed by scanning electron microscopy (SEM) and X-ray diffraction (XRD). The CMC-Ser-ACP nanocomplex solution could be preserved without any precipitation for 45 days. After the application of NaClO and through the guidance of Ser, ACP nanoparticles transformed into relatively orderly arranged hydroxyapatite (HAP) crystals, generating an aprismatic enamel-like layer closely integrated with the demineralized enamel, which resulted in enhanced mechanical properties for the treatment of early enamel caries lesions. The CMC-Ser-ACP nanocomplex solution is a remineralization system with great solution stability, and when NaClO is added, it can rapidly regenerate an aprismatic enamel-like layer in situ on the demineralized enamel surface. This novel remineralization system has stable chemical properties and can greatly increase the therapeutic effects against early enamel caries.

**Graphical Abstract:**

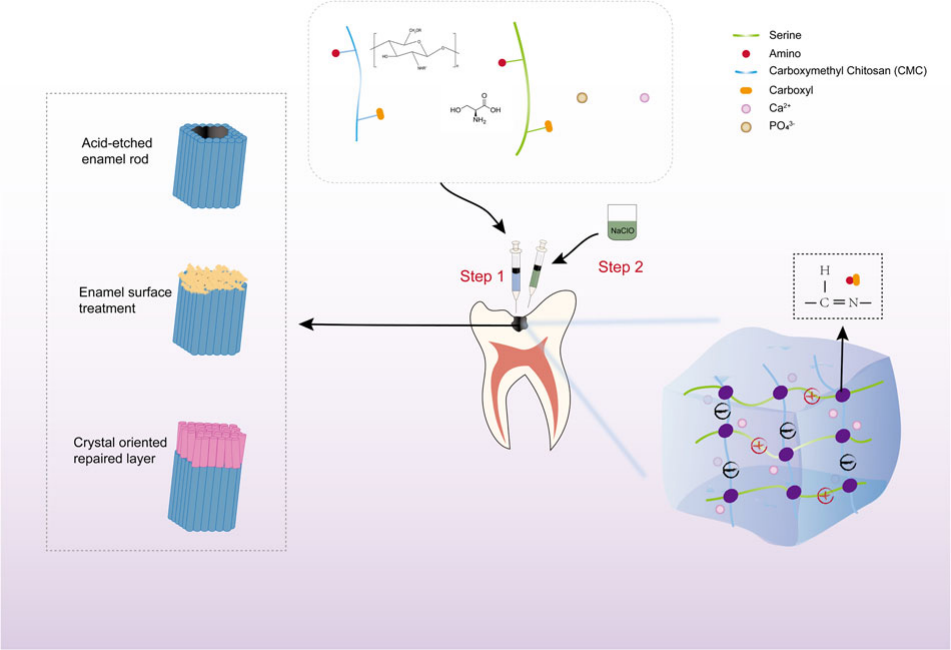

## Highlights

- Introducing Ser into CMC-ACP nanocomplexes improved their stability for up to 45 days.
- NaClO (1%) accelerated the process of transforming ACP into HAP crystals.
- CMC-Ser-ACP nanocomplexes regenerated aprismatic enamel-like crystals.
- Simpler preparation process proposed for CMC-Ser-ACP nanocomplexes.

## Introduction

Early enamel caries is one of the leading diseases among millions of children and adolescents [1] and has become a more critical research topic in cariology. Remineralization therapy is the primary approach for repairing enamel surfaces demineralized by acid, whether in foods or generated by bacteria. Demineralization results in an increase in the pore diameter of tooth hard tissues and a reduction in structural intensity, with minerals in a kind of unsaturated state [2]. It is essential to note that the outermost enamel layer is the most mineralized compared to other enamel layers, playing a unique role in preventing prismatic structural damage caused by acid attack. Early enamel caries mainly presents as white spot lesions (WSLs), with imbalanced calcium-phosphorus metabolism and a lack of carious cavities generation. Unfortunately, the effect of the inherent mechanism of calcium and phosphate ion redeposition in inhibiting demineralization is limited, indicating the need for optimal remineralization materials [3].

Hydroxyapatite (HAP) crystals are the key components of enamel and are generated by a transient amorphous precursor called amorphous calcium phosphate (ACP) [4]. In the enamel formation process, interactions between amelogenin (AMEL) and ACP are crucial for the stability of ACP and its HAP crystal transformation ability [5]. However, ACP loses its inherent remineralization ability by rapidly transforming into a more stable calcium phosphate (CaP) crystal in aqueous solutions. Hence, pure ACP solution is challenging to store and detrimental to enamel remineralization in the oral cavity [6]. Researchers have concentrated on utilizing noncollagenous protein analogs to stabilize ACP [7], such as polyaspartic acid [8], polyacrylic acid [9], poly(allylamine hydrochloride), and casein phosphopeptides (CPP) [10], which affect the inhibition of caries through ACP changes into HAP, blocking enamel pores and dentinal tubules. Nevertheless, the complex preparation protocols of these materials greatly limit their application.

One promising material for stabilizing ACP is carboxymethyl chitosan (CMC) [11], which interacts with calcium ions through electrostatic interactions to form a stable drug-loading nanogel system. Researchers used it to treat osteoporosis and found that the therapeutic effect was better than that of calcium carbonate [12]. CMC contains numerous carboxy groups and has strong electrostatic interactions with calcium ions, and it is also a biodegradable polysaccharide with ampholyte properties that can inhibit nanoparticle aggregation, which favors ACP nanoparticle stabilization in CMC-ACP nanocomposite solutions, so many researchers utilize CMC to stabilize ACP to achieve enamel remineralization [13]. However, according to our team’s previous studies [14], CMC has a poor performance in stabilizing ACP by itself, so we decided to explore a novel material based on CMC for stabilizing ACP, inducing its permeation into demineralized enamel in a stable liquid state with a better remineralization effect.

Another candidate substance is L-serine (Ser), an essential amino acid in organic mineral media that plays a significant role in the mineralization of enamel and dentin. In addition, the Ser-16 phosphorylation site is the only confirmed key site that regulates mineralization in vitro [15]. The P148 site on AMEL (~20 kDa) contains the Ser-16 phosphate group, which significantly inhibits calcium phosphate deposition by preventing its transfer into crystals [16]. Furthermore, dentin sialophosphoprotein (DSPP) participates in the mineralization of enamel, and Ser is a crucial amino acid in DSPP, accounting for 45%~50% of its amino acid sequence [17]. In addition, negatively charged Ser strongly binds with Ca^2+^ in the solution, and it exists as a zwitterion under acidic conditions. The larger number of amino and carboxyl groups also enables it to bind to ACP more tightly [18]. It has been reported that amino acids can reduce the nucleation activation energy, increase the crystal nucleation density, change the spatial structure, increase the spatial effect, and generate a crystal structure [19]. Inspired by this information, it is speculated that Ser can induce the growth of crystals on the surface of enamel.

It is difficult to precisely and highly efficiently regenerate enamel-like mineral crystals on the demineralized enamel surface. The innovative concept of remineralization therapy involves creating a biomimic material that has a diameter less than that of the dentinal tubule to permeate into it easily and then rapidly generate orderly aligned mineralized crystals. Based on the above statements, we hypothesized that CMC and Ser cooperate to stabilize ACP and that a CMC-Ser-ACP nanocomplex solution could be developed via the isoelectric point property of CMC and Ser, the ampholyte property of CMC, and the strong electrostatic interactions between Ser and Ca^2+^. Then, CMC, Ser, and ACP would be distributed uniformly in the whole solution. Moreover, we explored an effective way to trigger the phase transition of the CMC-Ser-ACP solution, allowing it to cover demineralized enamel stably and quickly and potentially blocking enamel pores, while avoiding strict reaction conditions and highly immunogenic chemical substances, creating an effective and promising remineralization method over early enamel caries and providing novel ideas for clinical application.

## Materials and methods

### Materials

Ser (L-serine) with a molecular weight of 105.09 Da was purchased from MACKLIN Co., Ltd (China). CMC (95%) was obtained from Honghai Co., Ltd (China). HAP was obtained from Sigma‒Aldrich Co., Ltd. Monobasic potassium phosphate (K_2_HPO_4_) was obtained from Sangon Biotech Co., Ltd (China). Calcium chloride dihydrate (CaCl_2_**·**2H_2_O) was obtained from Fuchen (China). Etch gel (35%) was obtained from Gluma Co., Ltd (Germany). NaClO (1%) was obtained from Longly Co., Ltd (China).

### Preparation and characterization of the remineralization solution

#### Preparation of the remineralization solution at room temperature (25 °C)

First, 35.4 mg of CaCl_2_·2H_2_O powder was added to deionized water and wholly dissolved by a magnetic stirring apparatus to obtain a CaCl_2_ solution at 6 mmol/L. Second, 100 mg CMC powder and 21 mg Ser were added to 20 ml deionized water in sequence and completely dissolved by a magnetic stirring apparatus to obtain the CMC-Ser solution with concentrations of 4.6 mmol/L CMC and 10 mmol/L Ser. In addition, 42 mg Ser and 25.2 mg K_2_HPO_4_ were sequentially added to 20 ml deionized water and completely dissolved, and CaCl_2_ solution was dropped into it under magnetic stirring to obtain the Ser-ACP solution with concentrations of 3 mmol/L K_2_HPO_4_, 6 mmol/L CaCl_2_ and 10 mmol/L Ser. Instead of 42 mg Ser, 100 mg CMC was used with the same protocol to prepare the CMC-ACP solution with concentrations of 4.6 mmol/L CMC, 3 mmol/L K_2_HPO_4_ and 6 mmol/L CaCl_2_, and the pH value was adjusted to 7.0. Finally, 100 mg CMC, 42 mg Ser, and 25.2 mg K_2_HPO_4_ were successively added to 20 ml deionized water, the solution was stirred until the components were completely dissolved, CaCl_2_ solution was added dropwise, and the pH value was adjusted to 6.5 to obtain the final CMC- Ser-ACP solution with concentrations of 4.6 mmol/L CMC, 3 mmol/L K_2_HPO_4_, 6 mmol/L CaCl_2_ and 10 mmol/L Ser. The speed of the magnetic stirring apparatus was set at 1500 rpm.

#### Remineralization solution characterization

The morphological characterization of the remineralization solution was performed by TEM using JEOL JEM-1230 equipment. The samples were examined by dropping them into the corresponding carbon support film, desiccating the water from the 400-mesh copper grid, and then allowing the samples to air-dry. The physicochemical properties were determined by FTIR. The samples were freeze-dried at a temperature of −80 °C and a pressure of less than 10pa for 12 h to obtain floccules in the solid state. The changes in the chemical bonds and functional groups in CMC and Ser were tested, and the interactions were analyzed. The zeta potential of the samples was measured by a Zeta Sizer Nano ZS90 apparatus at a wavelength of 488 nm and an angle of 173°.

### Enamel sample preparation

Ten extracted teeth without cracks, caries, or other defects were acquired from orthodontics extraction patients. First and foremost, all patients who understood and agreed to this research were willing to sign a written informed consent form. Every tooth was cut into four enamel slices, with dimensions of approximately 4 mm × 4 mm × 1 mm (length × width × height), and these enamel surfaces were polished with 200, 400, 800, 1000, 1200, 1600, and 2000 grit abrasive paper in sequence under running water. All samples were ultrasonicated with deionized water for 30 min to remove the smear layer, cleaned with 75% ethanol and sealed with water-resistant nail varnish, reserving a 2 mm × 2 mm window for follow-up work. The procedures and methods were approved by the Medical Ethics Committees of Tianjin Medical University(TMUaMEC 2022023).

### Remineralization of the demineralized enamel model

Forty enamel samples were randomly split into four groups. Group A consisted of sound enamel without any treatments. Group B was etched with 35% etch gel to obtain demineralized enamel samples as the control group. The demineralized samples were prepared before remineralization treatment in Groups C and D. The samples in Groups C and D were evenly brush-coated with CMC-ACP solution and CMC-Ser-ACP solution, and then 1% NaClO was applied to the treated surface for 3 s, twice a day for ten consecutive days [19]. Four group samples were dehydrated using graded ethanol, 50%, 70%, and 90% ethanol sequentially for 20 min and then 100% ethanol for 1 h.

### Characterization of the remineralized enamel samples

SEM analysis was performed to examine the surface morphology of the dehydrated samples randomly selected from the four groups. The samples were placed on a conductive adhesive, sputter-coated with gold, and observed with a voltage of 10 kV by FE-SEM using Zeiss Gemini 300 equipment. Using FE-SEM S-4800, the enamel samples of the CMC-Ser-ACP treatment group were observed, and the element constituent ratio and distribution of the newly generated crystals were analyzed.

### XRD

Before powder XRD analyses, the dried samples were manually ground in a mortar using a pestle. XRD experiments were performed (XRD-6000X-ray diffractometer, SHIMADZU, Tokyo, Japan) in a scan pattern with a Rigaku diffractometer using a Cu Ka radiation source (*k* = 1.542 Å) scanning at 70 Kv and 50 mA in the 2*θ* range of 0°~60°.

### Statistical analysis

The experiments were repeated three times to assure the validity of the results, and all data are expressed as the means ± standard deviations (SDs). Significant differences were identified by performing one-way ANOVA, and Student–Newman‒Keuls (SNK) tests were used for multiple comparisons. All data are presented as the means ± standard deviations (${\bar{\mathrm{X}}} \pm {\mathrm{SD}}$), and a value of *P < 0.05 was used to indicate significant differences. All statistical data analyses were calculated by SPSS 23.0 software.

## Results

### TEM

**Fig. 1:**
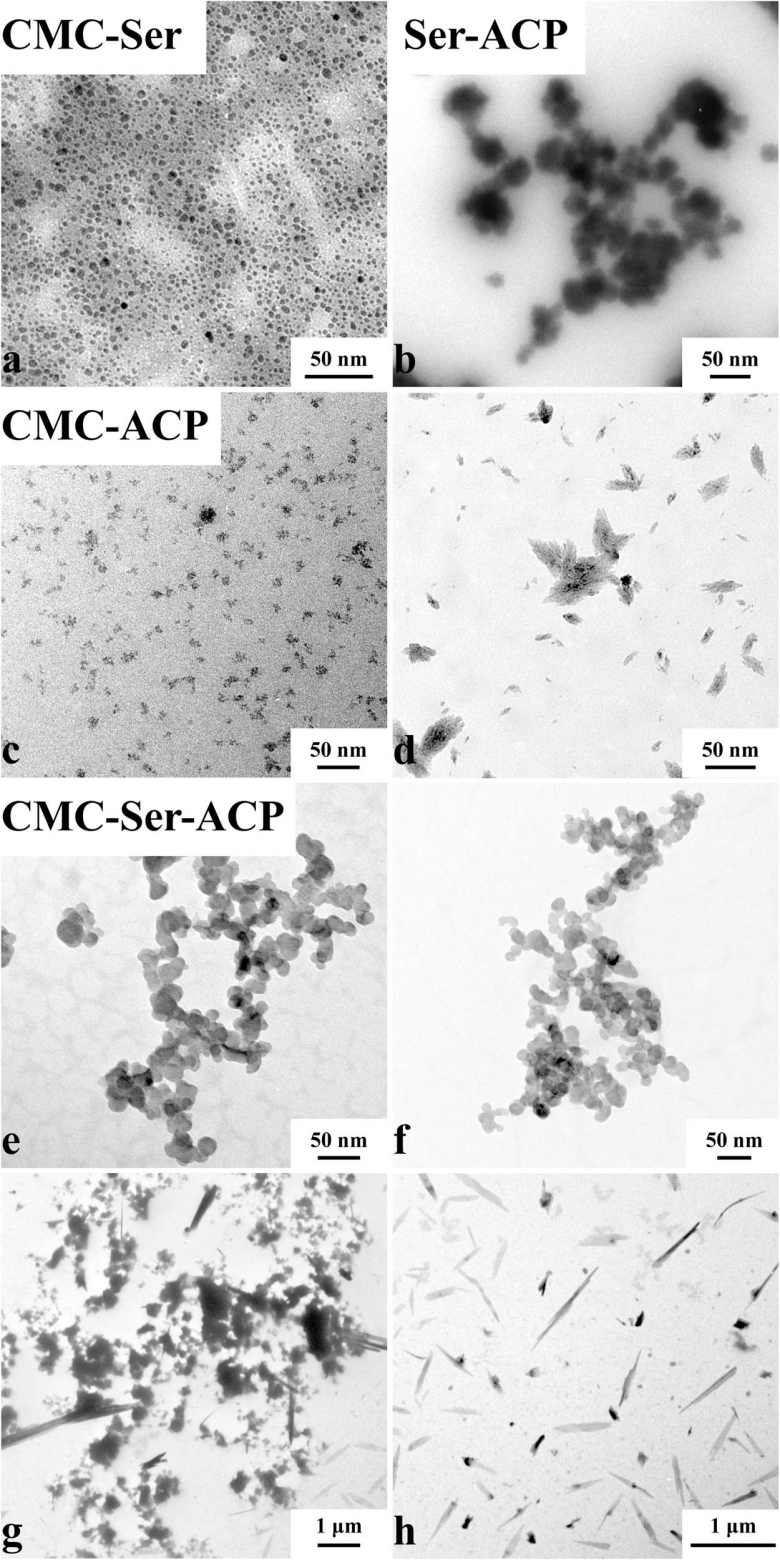
TEM characterization of samples. **a** Morphology of the CMC-ACP solution. **b** Morphology of the CMC-Ser-ACP solution. **c** Morphology of the CMC-ACP solution on the 1st day. **d** Morphology of the CMC-ACP solution on the 15th day with tiny, mineralized crystals formed. **e**, **f** Morphology of the CMC-Ser-ACP solution on the 1st and 45th days. The ACP nanoparticles were stable in the solution without any precipitation. **g** Morphology of the CMC-Ser-ACP solution on the 50th day with a few crystals formed. **h** Morphology of the CMC-Ser-ACP solution on the 60th day with large plate-like crystals formed

**Fig. 2:**
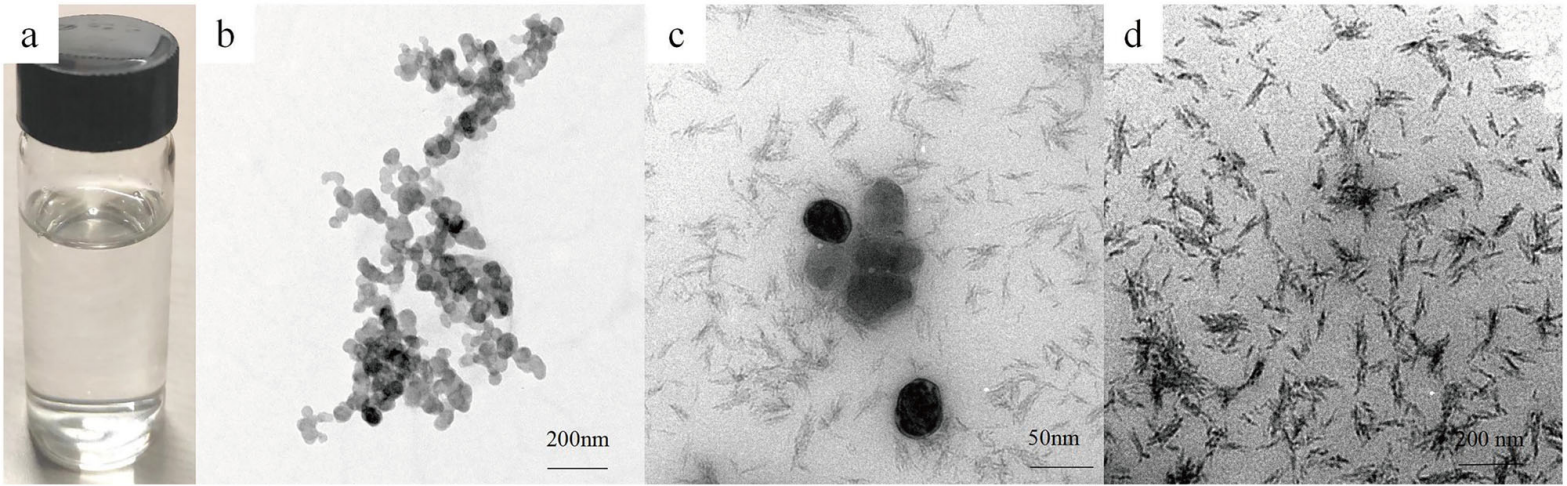
Digital photograph and TEM characterization of the CMC-Ser-ACP solution. **a** Digital photograph of the pellucid CMC-Ser-ACP solution. **b** TEM image of the CMC-Ser-ACP solution showing regular and sharply demarcated interconnected nanoparticles. After adding 1% NaClO (**c**, **d**), the one-day morphology showed that a mass of typical mineralized crystals formed

TEM images of the CMC-Ser nanoparticles showed a spherical shape, with a diameter ranging from 5 to 15 nanometers, and exhibited a uniform distribution (Fig. 1a). The Ser-ACP nanoparticles (Fig. 1b) had irregular spherical shapes, and their boundaries were obscure; they were 50–200 nanometers in diameter and distributed in clusters. On the first day, the CMC-ACP nanoparticles (Fig. 1c) were found to have obscure boundaries, vary greatly in size, and have the loosest assembly pattern. They formed tiny, mineralized crystals on the fifteenth day (Fig. 1d). Nevertheless, the CMC-Ser-ACP nanoparticles (Fig. 1e) had denser and sharply demarcated spherical shapes, and the particle diameters were similar to each other, approximately 40 nm. These nanoparticles were stable in the clarified solution with no sediments after 45 days (Fig. 1f). However, they formed a few crystals on the 50th day (Fig. 1g). As time passed, large plate-like crystals formed, with micron-sized diameters, and a mass of sediments was generated in the solution (Fig. 1h). Figure 2a shows a digital photograph of the clarified CMC-Ser-ACP solution on the 45th day, while the CMC-Ser-ACP solution showed regular and sharply demarcated interconnected nanoparticles (Fig. 2b). The one-day morphology after the addition of 1% NaClO (Fig. 2c, d) showed that a mass of typical mineralized crystals formed.

### FTIR

**Fig. 3:**
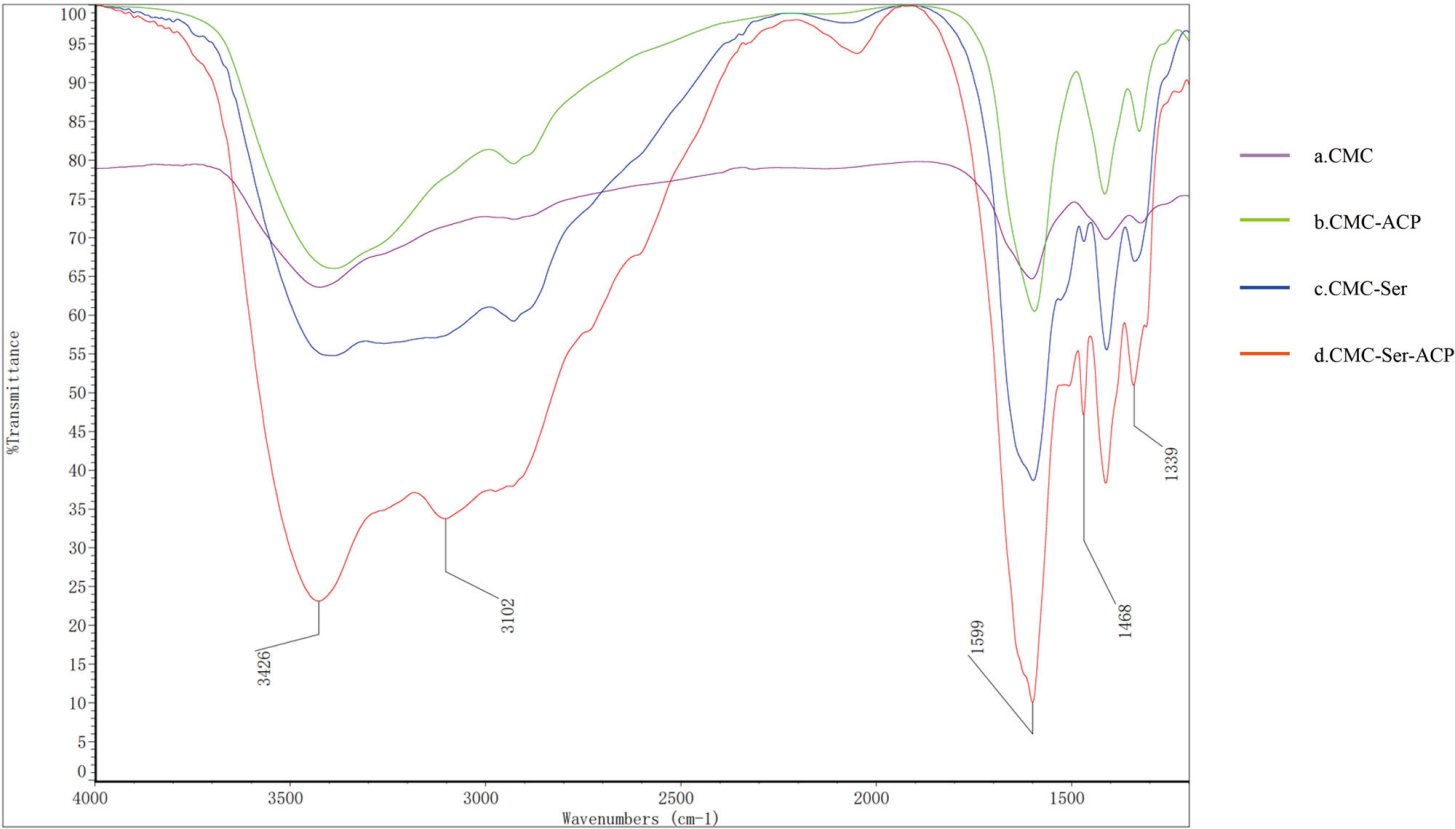
FTIR analysis of the four group samples. CMC solution (**a**), CMC-ACP solution (**b**), CMC-Ser solution (**c**) and CMC-Ser-ACP solution (**d**). FTIR spectra showing typical characteristic peaks of C=O, N–H, and C–N of the CMC-Ser-ACP solution (**d**), indicating the formation of amide bonds

Figure 3 shows the IR spectra of the four group solutions, in which there are apparent differences in the wavelengths and intensities of the peaks, especially regarding the C=O absorption peak at 1599 cm^−1^ and the N–H absorption peak at 3426 cm^−1^ (Fig. 3a–c). After introducing Ser into the CMC-ACP solution (Fig. 3d), the N–H absorption peak at 3426 cm^−1^, the C=O absorption peak at 1599 cm^−1^, the N–H bending vibration absorption peak at 1468 cm^−1^ and the C–N absorption peak at 1339 cm^−1^ were observed, representing the formation of amide bonds. Additionally, a new unsaturated carbon C–H absorption peak emerged at 3102 cm^−1^, indicating that new =CH_2_ groups formed. These results demonstrated that multiple covalent bonds formed between CMC and Ser with enhanced Ca^2+^ chelation to produce a marked effect on ACP stability.

### Zeta potential

The zeta potentials of the CMC solution (Fig. S1a) and L-Ser solution (Fig. S1c) were approximately −38.5 mV and −27.3 mV, respectively. These two solutions were negatively charged when they stabilized ACP (Fig. S1b, d), and their zeta potentials were approximately −11.6 mV and −6.1 mV at pH 7.0. However, when they cooperated to stabilize ACP (Fig. S1e), the zeta potential of the CMC-Ser-ACP solution was approximately −19.3 mV at pH 6.5. Indeed, when the absolute value of the zeta potential was more than 15 mV, the solution tended to be stable.

### XRD

**Fig. 4:**
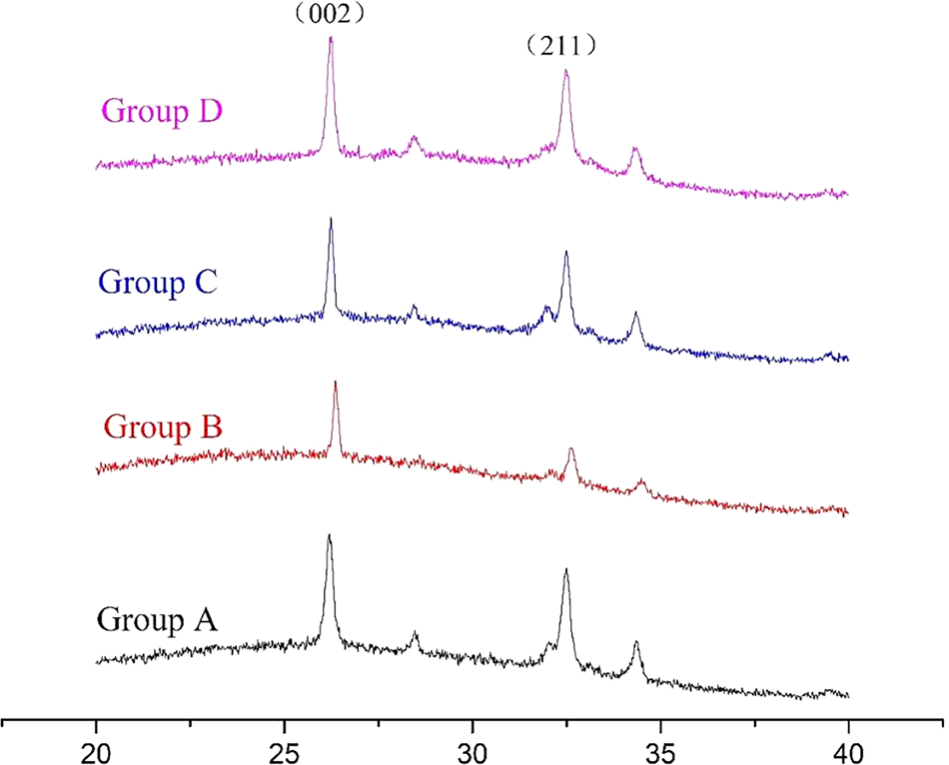
XRD spectra of the enamel samples, in which the representative HAP peak is marked. A: sound enamel group; B: acid-etched enamel group; C: CMC-ACP solution-treated group; D: CMC-Ser-ACP solution-treated group

Figure 4 shows four peaks in the X-ray diffraction pattern denoting the mineral facies of various enamel samples. The 2θ values of the diffraction peaks of (002) and (211) were 25.78° and 31.84°, respectively, representing the characteristic XRD peaks of HAP crystals in the sound enamel group (A). The intensities of the diffraction peaks significantly decreased in the acid-etched group (D), suggesting that the HAP crystals were severely damaged on the surface of the enamel. Additionally, the diffraction peaks of (002) and (211) formed by CMC-ACP nanoparticles (Group C) were more intense than those of the acid-etched group but less intense than those of the sound enamel group. Interestingly, the diffraction peaks generated by CMC-Ser-ACP nanoparticles (Group D) were similar to those of the sound enamel group, demonstrating a better remineralization effect. In general, the ratio between the (002) and (211) diffraction peaks represents the orientation degree of HAP crystals along the c axis, that is, the consistency of the remineralized HAP crystal growth direction. Table 1 shows the ratio of diffraction peaks (002) and (211) in the four groups, indicating that the HAP crystal growth direction of demineralized enamel was different from that of sound enamel, and a limited change was observed even when CMC-ACP solution was applied to the surface of acid-etched enamel. Fortunately, we detected that remineralized crystals had a similar orientation degree along the c axis in sound enamel after introducing Ser into CMC. Thus, we can speculate that Ser can guide ACP to generate remineralized crystals that resemble sound enamel.

**Table 1:** The ratio of XRD (002)/(211) diffraction peaks among the A, B, C, and D groups

	Group A (*)	Group B	Group C (*)	Group D (*)
(002)/(211)	0.83 ± 0.20	1.12 ± 0.14	1.07 ± 0.06	0.81 ± 0.20

*n* = 4, mean ± SD, *P < 0.05, compared to group B

### SEM

**Fig. 5:**
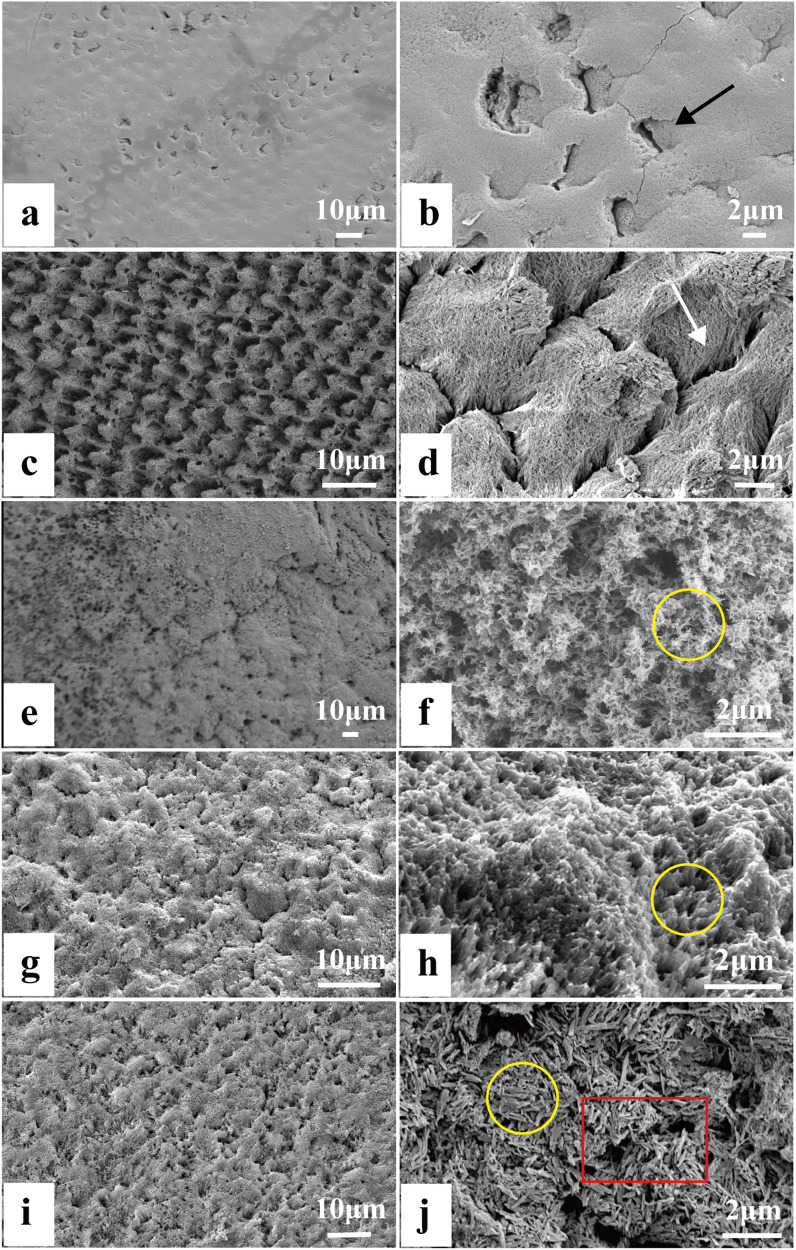
SEM image showing the surface morphology of sound enamel in (**a**, **b**). Fish-scale surface morphology of demineralized enamel (**c**, **d**). Acid-etched enamel soaked in artificial saliva for 2 weeks, with small and irregular remineralized crystals (**e**, **f**). Acid-etched enamel treated by CMC-ACP solution and NaClO, with less continuous remineralized crystals (**g**, **h**). Acid-etched enamel treated with CMC-Ser-ACP solution and NaClO (**i**, **j**) forming a dense mineral layer with relatively aligned remineralized crystals. The black arrow and white arrow indicate the pores in sound enamel and demineralized enamel, respectively. The yellow labels indicate the typical newly formed remineralized crystals in each experimental group

The surface morphology of the sound enamel included a layer of rodless enamel with only a few pores; the rest of the layer were in a closed state (Fig. 5a, b). The demineralized enamel showed some prism structures, such as fish-scale structures, with plenty of larger pores (Fig. 5c, d). However, after soaking the demineralized enamel in artificial saliva for two weeks, a remineralized layer was observed on the surface of the enamel, but the mineralized crystals were tiny and irregularly aligned (Fig. 5e, f). Furthermore, we applied CMC-ACP solution and NaClO solution to the acid-etched enamel in sequence, generating a thin-layer remineralized structure, piled with each other, but the newly formed crystals had poor continuity (Fig. 5g, h). However, when we merely swapped the CMC-ACP solution for the CMC-Ser-ACP solution in the previous step (Fig. 5i, j), a denser layer of remineralized crystals aligned in an orderly manner was observed, which was similar to the natural enamel prism structure.

To further verify the remineralization effect of the CMC-Ser-ACP solution, we randomly chose a remineralized surface, namely, the red box area in Fig. 5j, and analyzed the distribution of the main elements by EDX (Fig. S2). We found that its ratio of calcium and phosphorus (Table. S1) was similar to that of sound enamel (1.67), indicating that the CMC-Ser-ACP solution had a better remineralization effect. Figure S3 shows the surface distribution of the main elements, C, O, S, Ca, P, and N, which were consistently distributed on most basal surfaces, confirming the combination of CMC and Ser.

## Discussion

HAP crystals are the most stable existence mode of enamel under physiological conditions, and their integrity is maintained by the saliva homeostatic mechanism through normal calcium phosphate metabolism involving statherin and proline-rich protein. However, hard tissue cannot be repaired by saliva if this mechanism is impeded, and tooth decay ultimately occurs. Organic macromolecules are trace constituents of enamel and consist of abundant functional groups, such as –COO^−^ anions and –NH_3_^+^ cations, which can interact with inorganic ions and induce the crystalline process [20]. Thus, it is crucial to utilize functional organic matter to guide ACP precursors to efficiently generate HAP crystals in vitro.

It has been reported that ACP is an excellent precursor for HAP, although it can rapidly transform into a stable CaP state in aqueous solution, so it is difficult to use ACP to remineralize WSLs in the oral cavity directly unless we effectively stabilize ACP. According to our research team’s previous and present studies [19, 21], CMC can be used to stabilize ACP to generate CMC-ACP nanocomplexes with good solution stability in a week. In this study, after introducing Ser into CMC, the nanocomplex solution stabilization ability increased more than sixfold. CMC is a long-molecular-chain polymer with extraordinarily controllable release properties in drug delivery systems [22]. This long chain structure can wrap around the surface of calcium ions to inhibit their combination with phosphate ions and thus prevent the formation of CaP crystals [23]. In general, ACP generates randomly aligned plate-like HAP crystals in 24 h. After adding CMC into ACP solution with high concentrations of calcium and phosphate [15], the solution sustained transparency without any sediments and could be preserved for approximately one week [19]. Nevertheless, just one week of solution stability is far from the minimum requirement for rapid remineralization therapy in clinical applications. Therefore, in this study, we used 6 mmol/L Ca^2+^ and 3 mmol/L PO_4_^3−^ to stabilize ACP. The TEM, FTIR, XRD, and FE-SEM results strongly suggested the ability of CMC to stabilize ACP (Fig. 1c, d), including ACP nucleation inhibition, precipitation, and phase transformation.

AMEL plays a crucial role in enamel crystallinity as an entity structure participating in enamel growth [24, 25]. Accordingly, 20-kDa AMEL P148 can significantly inhibit the homogeneous nucleation of ACP nanoclusters in vitro, preventing them from transforming into crystals. This biological effect has been reported concerning monophosphate functional groups and the hydrophilic carbon terminal (C-terminal) [15]. It is postulated that Ser could be a potential stabilizing agent to inhibit the phase transformation of ACP and prolong the induction period of ACP. Consequently, in this study, nanocomplexes of CMC-Ser-ACP were prepared at room temperature, avoiding organic solvents and harsh reaction conditions, such as high temperatures and pressures. Compared to CMC-ACP nanocomplexes, it remarkably extended the stable state of ACP (Fig. 1d, f), inducing ACP mineralization in vitro and generating more gigantic plate-like crystals, further enhancing the remineralization effect of WSLs of early enamel caries. All these advantages favor its future clinical application.

Early enamel caries is characterized as severely damaged enamel subsurface compared to the outermost layer of enamel, with a porous structure but a relatively intact surface layer. It has been verified that acid solution is beneficial for the deep mineralization of enamel because it can penetrate the lesions of comparatively integrated early enamel caries [26]. However, if the solution pH is too low, enamel tends to undergo subsurface demineralization. Therefore, considering the characteristics of Ser under acidic conditions, the pH value of the CMC-Ser-ACP solution was selected as 6.5.

Because different concentrations of amino acids exert different effects on mineralization, they induce mineralization at a low concentration but inhibit mineralization at a high concentration. Before formal experiments, we performed a preliminary experiment that confirmed that the optimal concentration of Ser and CMC for collaborative stabilization of ACP was 10 mmol/L. It was reported that unphosphorylated full-length recombinant amelogenin rP172 could prolong the ACP induction period [27], in which tiny ACP nanoparticles were stabilized and aligned as linear needle-like particles and subsequently transformed into HAP crystals [28]. While this process was identified with the phenomena observed by TEM in this experiment, we discovered that the CMC-Ser-ACP solution was transparent and without any precipitation for 45 days (Fig. 2a), and then the color of the solution gradually became blanched and turbid until the mineral crystals were complete formed at the 60th day (Fig. 1h). This verified the ability of Ser to stabilize ACP nanoparticles effectively. Additionally, by conducting TEM characterization over time, we can clearly observe the occurrence of crystallization and ultimately determine the stability duration of these nanocomplexes.

In the spontaneous generation period of enamel, inhibition of the crystal nucleation effect may occur during the secretion phase of enamel generation, which prevents unnecessary crystal generation from numerous extracellular enamel matrices. During this time, tiny enamel crystals formed, accounting for 10–20% of the extracellular matrix (ECM), and the rest of the ECM components were proteins and water [29, 30], which could be indicative of the enamel mineralization regulatory mechanism of Ser. The molecular simulation indicates that the bond between COO^−^ and Ca^2+^ was the crucial chemical bond that allowed COO^−^ at the molecular C-terminal domain compactly bond to the (001) lattice plane [31, 32]. Ser exists as a zwitterion with free COO^−^ and –NH_3_^+^ as terminal groups in acidic conditions [18], and adding Ser into CMC could greatly increase the amount of COO^−^, allowing it to more tightly adsorb onto HAP crystals. The FTIR results indicated that the C=O absorption peak and N–H absorption peak underwent bathochromic shifts (Fig. 3c), which verified the increase in –COO^−^ anions and –NH_3_^+^ cations, respectively. Figure 3d shows the formation of amide bonds, which could undergo hydrogen bonding interactions intramolecularly or with water molecules [33]. Thus, we suggested that the higher the number of newly formed hydrogen bonds in solution is, the better the solution stability, which could offset the repulsion between identical charges among the terminal groups to increase the adsorption density. Moreover, Ser may induce oriented remineralization through the tight bonding of the –COO^−^ anions to the (001) lattice plane of HAP crystals.

CMC and Ser were negatively charged in the solution because their isoelectric point was lower than the solution pH, and the repulsive force between them allowed CMC to be highly dispersed (Fig. 1a). The TEM image revealed that CMC and Ser were uniformly distributed in a spherical structure at the nanoscale, indicating that a more stable solution system formed. When the zeta potential of the complexes was less than −3.5 mV, spherical nodules and aggregated spheres formed in the internal structure, leading to a further decrease in the zeta potential [34]. The zeta potential of ACP was approximately −1.1 mV, which was exemplified by its extreme instability and easy precipitation in aqueous solution [35]. Using CMC or Ser to stabilize ACP resulted in an increase in zeta potential (Fig. S1b, d), indicating that both of them can be adsorbed on the ACP surface to inhibit ACP aggregation. The calcium-phosphorus ratio in CMC-Ser-ACP solution was 2:1; with abundant free Ca^2+^ in the solution, CMC and Ser adsorbed more tightly onto the ACP surface through electrostatic attraction. Therefore, the reason why the solution system became more stable was because CMC-Ser-ACP itself had a more stable structure, and the adsorption of CMC-Ser onto the ACP surface prevented it from responding to free Ca^2+^. Hence, when CMC-Ser-ACP nanoparticles made contact with the enamel surface, the negatively charged CMC-Ser was attracted by positively charged HAP crystals, and then they released calcium and phosphate, generating a crosslinked nanocoating to achieve enamel remineralization [3].

According to the AMEL regulatory mechanism of enamel generation, mineral phase transformation usually occurs at the dephosphorylated or degraded regulatory point in the AMEL; meanwhile, calcium ions and phosphate ions undergo crystal phase transformation within a certain period and are rapidly transformed into HAP crystals [15, 36]. Based on biomineralization theory, we must ensure that the biomaterial can induce remineralization in a rapid response pattern. NaClO is a superior material that can be applied in the oral cavity directly at a low concentration of 1% as a root canal irrigation agent. Therefore, we used it as a powerful surfactant to realize degradation and reduce the stability of CMC. Indeed, NaClO is a potent oxidizing agent because it can break the β-(1,4) linkages of chitosan for depolymerization [37], causing the Ser and the long chain of CMC to rupture into nonfunctional fragments, precluding their ability to stabilize ACP and thereby accelerating the remineralization process. Consequently, we used NaClO as the catalyst in this study, and the TEM images (Fig. 2c, d) proved that the disordered ACP nanoparticles rapidly transformed into crystal clusters. When NaClO was applied to the demineralized enamel surface, the XRD results (Fig. 4d) showed that the newly generated prism-like crystals resembled natural enamel crystals in terms of morphological characterization.

In our study, the TEM results verified that the effect of CMC-Ser on stabilizing ACP was significantly superior to that of using each of them alone, initially generating uniform nanoparticles and ultimately generating 2 μm long crystal structures (Fig. 1). Additionally, after NaClO treatment in the experimental groups, the transformation from the amorphous phase to crystal phase was rapidly completed within 24 h. The XRD results (Fig. 4, Table 1) revealed that the diffraction peaks of Groups A and D were similar, demonstrating that the introduction of Ser into CMC can guide ACP nanoparticle assembly to generate enamel-like remineralized crystals. The SEM images (Fig. 5) further confirmed that the newly generated compact and ordered remineralized crystals in the CMC-Ser-ACP solution significantly blocked the demineralized enamel pores. Because of the highly aligned new HAP structure, the enamel lesions achieved excellent chemical stability and mechanical properties [31]. In addition, adding Ser into the CMC-ACP solution effectively prolonged the stable period and induced remineralization in situ, practically improving the efficiency of clinical ACP use. Clinicians could apply the CMC-Ser-ACP solution to replenish the calcium and phosphate in the oral cavity to enhance remineralization.

Based on the aforementioned results of our work, CMC-Ser-ACP solution is an excellent remineralization solution system with significantly prolonged solution stability time, which has preferable application prospects in the treatment of early enamel caries. Clinicians need only apply it to WSLs to guide the generation of bulky plate-like remineralized crystals without other time-consuming steps. This study provides a novel idea for rapid enamel remineralization in situ in the biomimetic mineralization field. We will continue to explore and optimize the performance of this novel material in future studies, aiming to achieve better treatment outcomes while minimizing the period of therapy. Also, future studies should further investigate the remineralization effect in vivo to confirm the veracity of the experimental results and further strengthen our conclusions.

## Conclusions

In this study, CMC and Ser collaboratively stabilized ACP to generate CMC-Ser-ACP nanocomplexes with great solution stability for 45 days, and ACP nanoclusters were modified by NaClO to rapidly linearly arrange to further generate aprismatic enamel crystal structures in situ on the demineralized enamel surface. Since CMC-Ser-ACP nanocomplexes practically remineralized demineralized enamel, it could be a promising biomimetic mineralization material for treating human early enamel caries with less operation difficulty and more treatment efficiency.

### Supplementary information

Supplementary Materials
