# Supplementary material for: L-serine combined with carboxymethyl chitosan guides amorphous calcium phosphate to remineralize enamel
Source: J Mater Sci Mater Med. 2023 Sep 2;34(9):45. doi: 10.1007/s10856-023-06745-z (PMC10474979; doi:10.1007/s10856-023-06745-z)
Supplement: Supplementary file 1 — Supplementary Materials [file 10856_2023_6745_MOESM1_ESM.pdf]

Supporting information for

L-Serine Combined with Carboxymethyl Chitosan Guides Amorphous Calcium Phosphate to Remineralize Enamel

Yinghui Wang[a,1], Shuting Zhang[a,1], Peiwen Liu[a,d,1], Fan Li[a], Xu Chen[a], Haorong Wang[a], Zhangyi Li[c], Xi Zhang[a], Xiangyu Zhang[a,*] ,Xu Zhang[a,b,*]

[*] Authors to whom correspondence should be addressed.

[1]These three first authors contributed equally to this work.

Affiliations

a School and Hospital of Stomatology, Tianjin Medical University, Tianjin 300070, China.

b Institute of Stomatology, Tianjin Medical University, Tianjin 300070, China.

c Department of Stomatology, the Fifth Central Hospital of Tianjin, No. 41, Zhejiang Road, Tanggu, Binhai New District, 300450 Tianjin, China.

d Department of stomatology, No.7 people's hospital of Zhengzhou, No. 17, Jingnan 5th Road, Economic and Technological Development Zone, Zhengzhou City, Henan Province 450003, China.

Corresponding author

*Address correspondence to Prof. Xiangyu Zhang (e-mail: xzhang04@tmu.edu.cn) or to Prof. Xu Zhang (e-mail: zhxden@gmail.com, zhangxu@tmu.edu.cn).

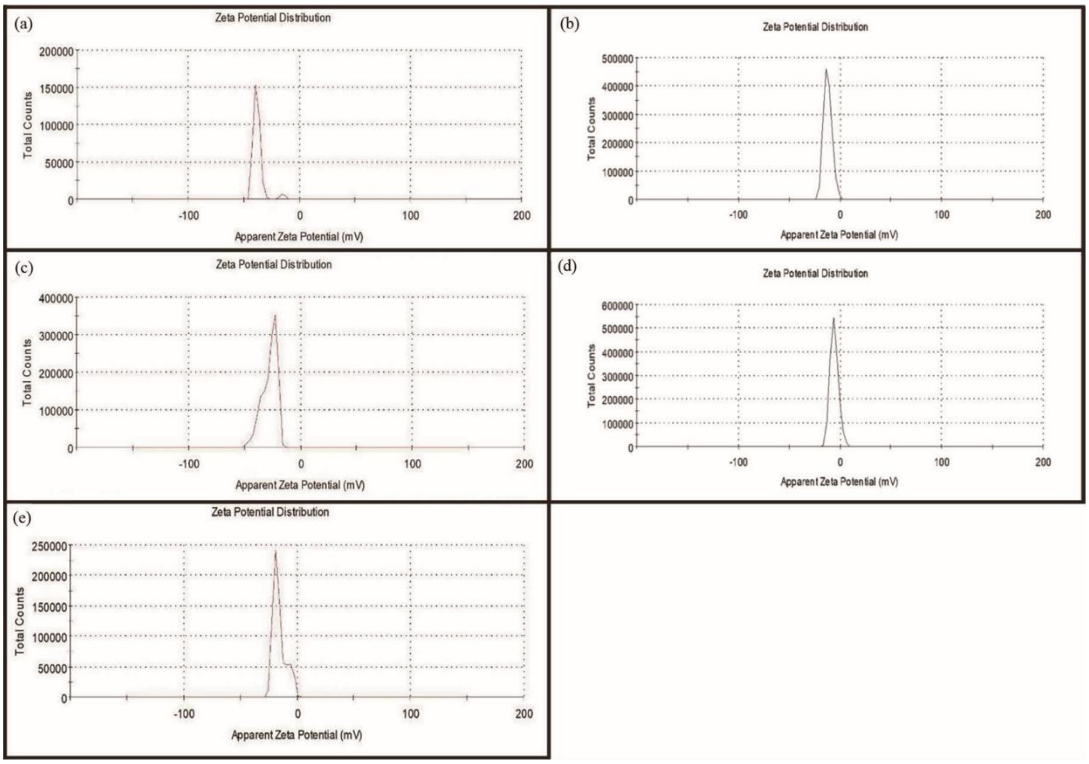

Fig. S1. Zeta potentials of the CMC solution (a), CMC-ACP solution (b), L-Ser solution (c), and Ser-ACP solution (d) at approximately -38.5 mV, -11.6 mV, -27.3 mV and -6.1 mV, respectively, and that of the CMC-Ser-ACP nanoparticles (e) at approximately -19.3 mV at pH 6.5.

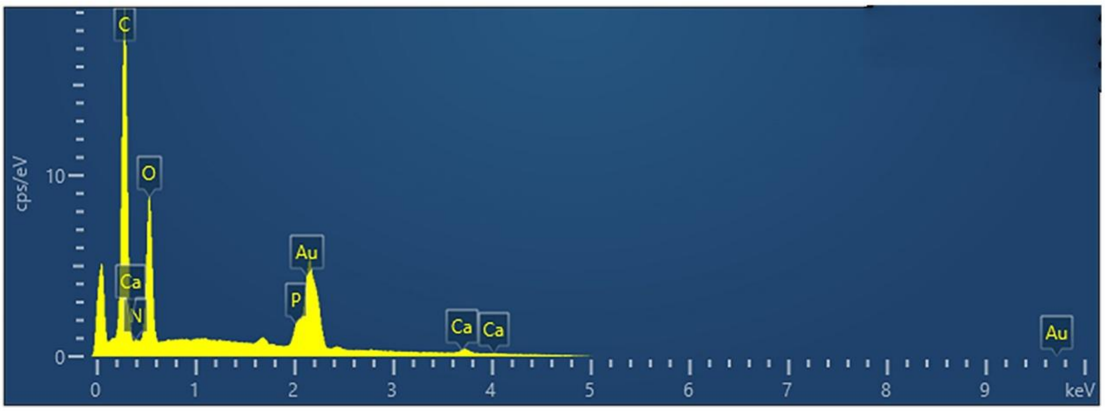

Fig. S2. EDX image of acid-etched enamel treated by CMC-Ser-ACP solution and NaClO.

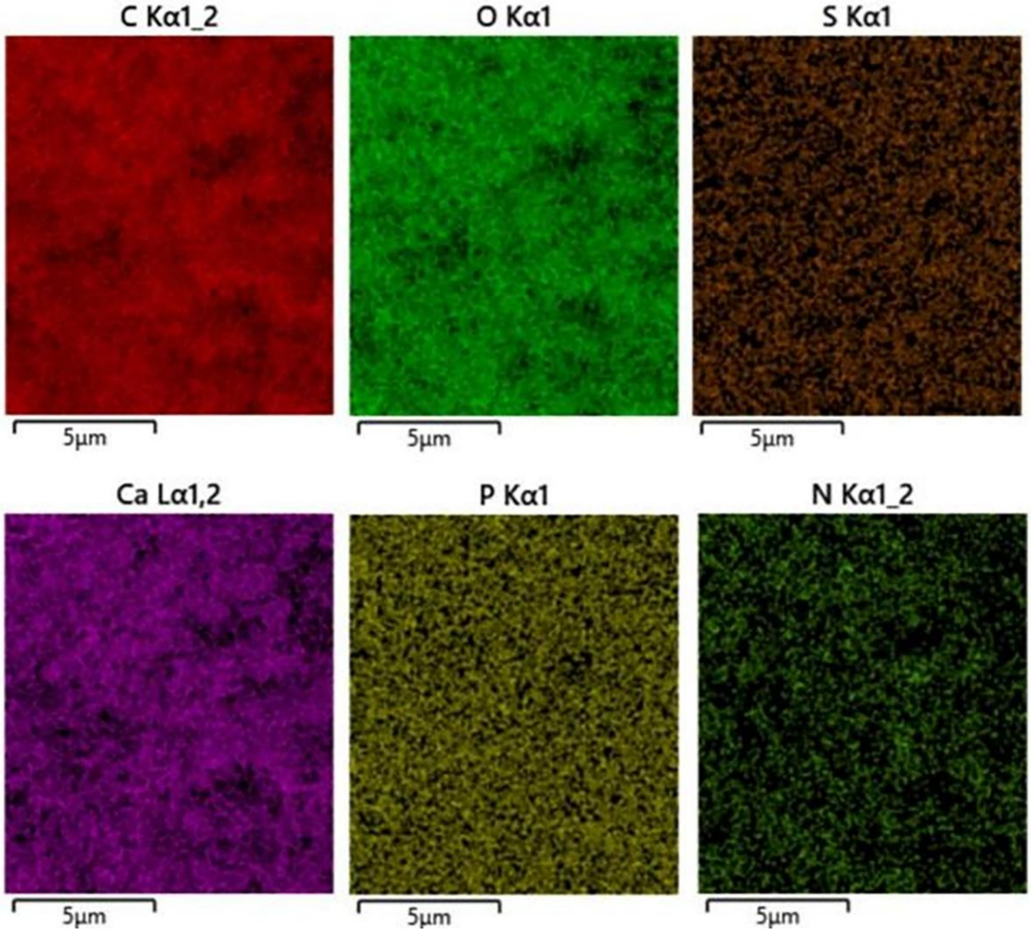

Fig. S3. Element distribution map of acid-etched enamel treated by CMC-Ser-ACP solution and NaClO.

Table S1.The ratio of main elements distribution of Acid-etched enamel treated by CMC-Ser-ACP solution and NaClO.

Element	Series	Weight%	Wt% Sigma	Atomic%
C	K-Series	24.32	0.30	61.51
O	K-Series	9.99	0.14	18.97
Au	M-Series	52.34	0.56	8.07
Ca	K-Series	8.44	0.64	6.40
P	K-Series	4.73	0.15	4.63
N	K-Series	0.19	0.17	0.42
Total		100.00		100.00
